# Traditional electrosurgery and a low thermal injury dissection device yield different outcomes following bilateral skin-sparing mastectomy: a case report

**DOI:** 10.1186/1752-1947-5-212

**Published:** 2011-05-28

**Authors:** Richard E Fine, Joshua G Vose

**Affiliations:** 1 WellStar Kennestone Hospital, 677 Church Street, Marietta, GA 30060, USA; 2 PEAK Surgical, Inc., 2464 Embarcadero Way, Palo Alto, CA 94303, USA

## Abstract

**Introduction:**

Although a skin- and nipple-sparing mastectomy technique offers distinct cosmetic and reconstructive advantages over traditional methods, partial skin flap and nipple necrosis remain a significant source of post-operative morbidity. Prior work has suggested that collateral thermal damage resulting from electrocautery use during skin flap development is a potential source of this complication. This report describes the case of a smoker with recurrent ductal carcinoma *in situ *(DCIS) who experienced significant unilateral skin necrosis following bilateral skin-sparing mastectomy while participating in a clinical trial examining mastectomy outcomes with two different surgical devices. This unexpected complication has implications for the choice of dissection devices in procedures requiring skin flap preservation.

**Case presentation:**

The patient was a 61-year-old Caucasian woman who was a smoker with recurrent DCIS of her right breast. As part of the clinical trial, each breast was randomized to either the standard of care treatment group (a scalpel and a traditional electrosurgical device) or treatment with a novel, low thermal injury dissection device, allowing for a direct, internally controlled comparison of surgical outcomes. Post-operative follow-up at six days was unremarkable for both operative sites. At 16 days post-surgery, the patient presented with a significant wound necrosis in the mastectomy site randomized to the control study group. Following debridement and closure, this site progressively healed over 10 weeks. The contralateral mastectomy, randomized to the alternative device, healed normally.

**Conclusion:**

We hypothesize that thermal damage to the subcutaneous microvasculature during flap dissection may have contributed to this complication and that the use of a low thermal injury dissection device may be advantageous in select patients undergoing skin- and nipple-sparing mastectomy.

## Introduction

Primarily because of inherent cosmetic and reconstructive advantages, the use of a skin- and nipple-sparing surgical technique during mastectomy is increasingly prevalent, especially for prophylactic mastectomy [1,2]. However, partial necrosis of the skin flap and/or nipple-areola complex is relatively common, affecting approximately 10% to 20% of patients [1-7]. Evidence suggests that thermal injury to the subcutaneous microvascular supply caused during electrosurgical dissection of the skin flap may be a contributing factor [1]. This complication represents a significant source of post-operative morbidity and a barrier to nipple preservation and optimal cosmetic outcome.

We are currently conducting the first prospective, randomized, controlled study to evaluate the use of the PEAK PlasmaBlade (PEAK Surgical, Inc., Palo Alto, CA, USA) in simple mastectomy without immediate reconstruction. The PlasmaBlade is a US Food and Drug Administration-cleared and CE-marked, low-temperature tissue dissection device that uses pulsed radiofrequency energy discharges in conjunction with a highly insulated electrode design to cut with simultaneous hemostasis and approximately 75% less depth of thermal damage than traditional electrosurgical (that is, "Bovie") devices [8-10]. Because the PlasmaBlade device is priced competitively with comparable advanced electrosurgery systems ($200 to $300), it represents a cost-effective treatment alternative. Traditional electrosurgery devices (about $20) are significantly less expensive. In this trial, the two operative sides of each bilateral simple mastectomy were randomized to either the PlasmaBlade or standard of care (SOC; scalpel and electrosurgery) treatment groups, allowing for a direct, internally controlled comparison of outcomes with the use of the two different surgical instruments. The primary and secondary endpoints for the study are total serous drain output, post-operative Visual Analog Scale pain scores and narcotic consumption. While the data concerning these endpoints are still being collected and analyzed, we report herein the sole incidence of skin flap necrosis as an adverse event in the control (SOC) group and discuss its implications with respect to the use of traditional electrosurgical devices during skin- and nipple-sparing mastectomy.

## Case presentation

A 61-year-old Caucasian woman with a 17.5 pack year history of smoking presented with recurrent right-sided ductal carcinoma *in situ *(DCIS) one year after lumpectomy, partial breast irradiation, and sequential treatment with tamoxifen and raloxifene. In addition to a right-sided mastectomy with sentinel lymph node biopsy, the patient chose a prophylactic left-sided mastectomy and declined reconstruction. After we obtained her informed consent, the patient was included in the comparative surgery clinical trial (ClinicalTrials.gov Identifier: NCT00943605). This study was approved by the institutional review board of WellStar Kennestone Hospital (Marietta, GA, USA) and is being conducted in accordance with all ethical standards for human clinical research. According to the randomization protocol, SOC was used for the left-sided (prophylactic) mastectomy and the PlasmaBlade was used for the right-sided (interventional) mastectomy with sentinel lymph node biopsy.

Anesthesia was induced, and the patient was prepared and draped in the usual sterile fashion. For the left-sided mastectomy, an elliptical, Y-V advancement flap-type incision was made laterally using a number 10 scalpel blade to minimize the risk of a dog-eared deformity resulting from a large deposit of subcutaneous fat. The tissue flaps were developed down to the muscle fascia with a traditional electrosurgical device set on Coag 35W (Valleylab E2516 Electrosurgical Pencil with E1551X standard stainless steel electrode and ForceTriad™ Generator; Valleylab, Inc., Boulder, CO, USA). Using the same instrument and settings, the breast was dissected off the chest wall in a mediolateral direction with the pectoralis fascia included. The remaining attachments of the breast to the serratus anterior were divided and followed superiorly to the base of the axilla. The incision was reapproximated using 5-0 and 4-0 polydioxanone sutures and 1-inch SteriStrip™ adhesive skin closures (3M, Inc., St Paul, MN, USA). Tegaderm™ transparent dressing (3M, Inc.) and Telfa™ Ouchless non-adherent dressing (Covidien, Inc., Mansfield, MA, USA) were used for the dressing.

Prior to incision of the right breast, 2mL of methylene blue diluted with 3mL of normal saline were injected into the subareolar region. The PEAK PlasmaBlade 4.0 with PULSAR Generator (PEAK Surgical, Inc.) was then used to create a standard elliptical skin incision (Cut 5: 2W) and to develop the subcutaneous dissection down to the muscle fascia (Cut 6 for dissection and Coag 7 for bleeding control). The breast was then dissected off the chest wall, and the incision was closed and dressed in a similar fashion to the left breast. The patient left the operating room in stable condition and was discharged to home the following morning. The total estimated blood loss from both sides was 50mL.

**Figure 1:**
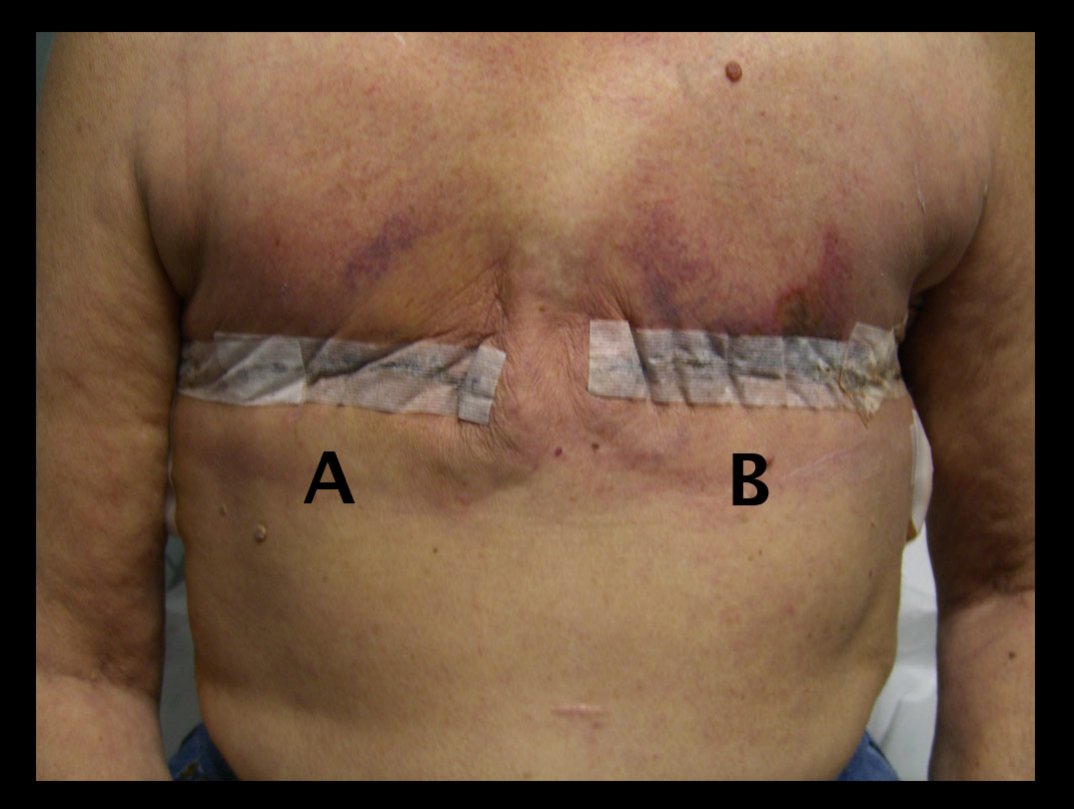
**Healing progress 11 days post-mastectomy**. **(A) **PlasmaBlade. **(B) **Standard of care (SOC; scalpel and traditional electrosurgery). Note increased erythema and ecchymosis on the SOC side.

**Figure 2:**
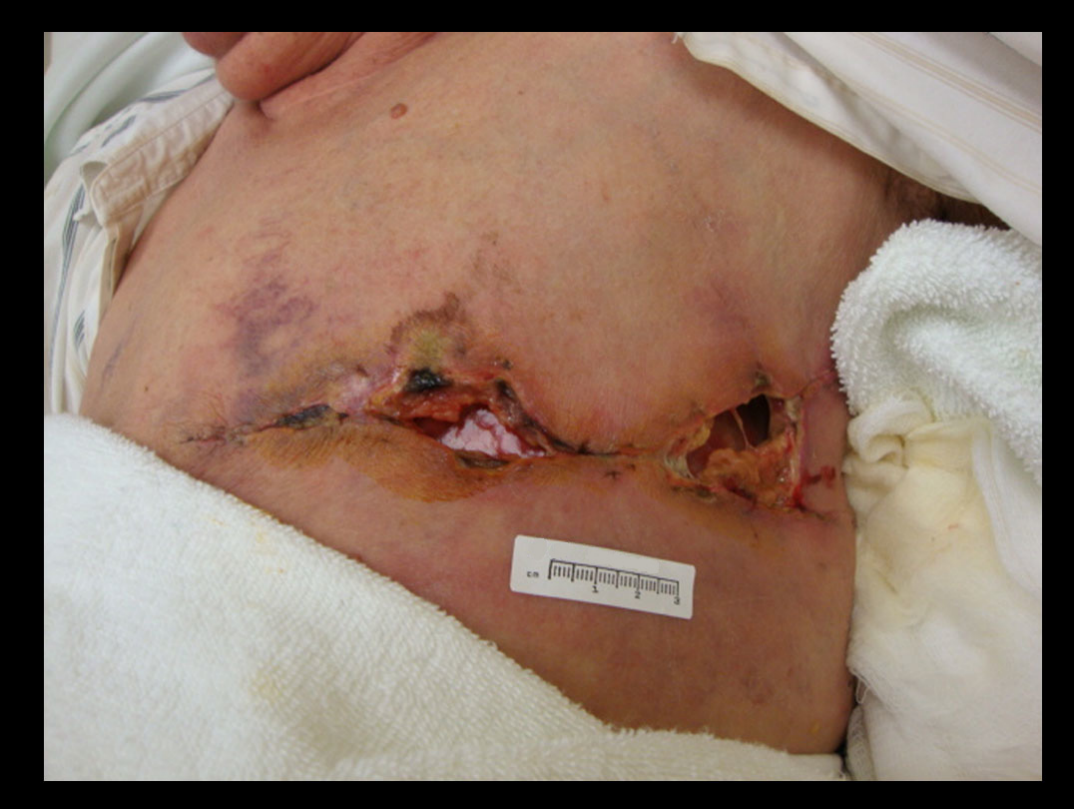
**Healing progress 16 days post-mastectomy**. Presentation of significant wound necrosis on the SOC side.

**Figure 3:**
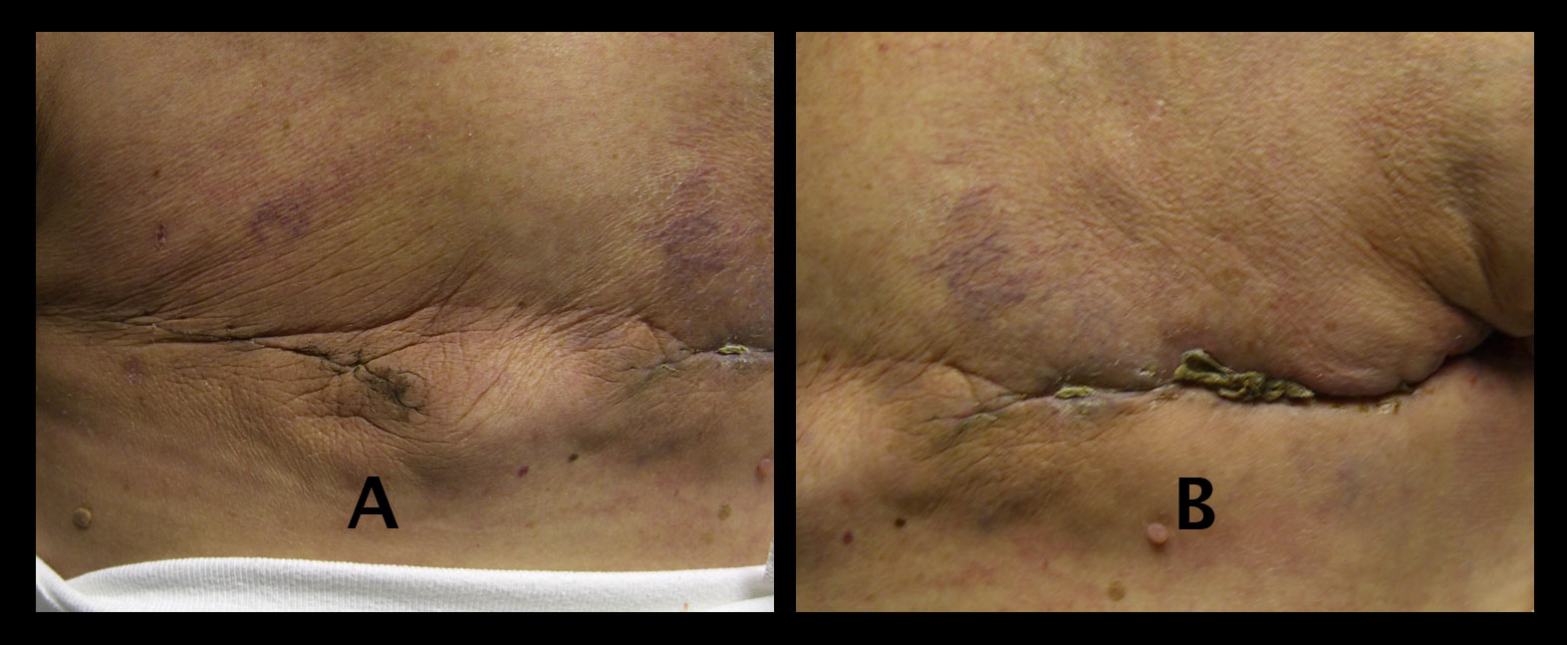
**Healing progress seven weeks post-mastectomy**. **(A) **Healed PlasmaBlade mastectomy. **(B) **Improved healing on the SOC side with noted small area of residual eschar.

**Figure 4:**
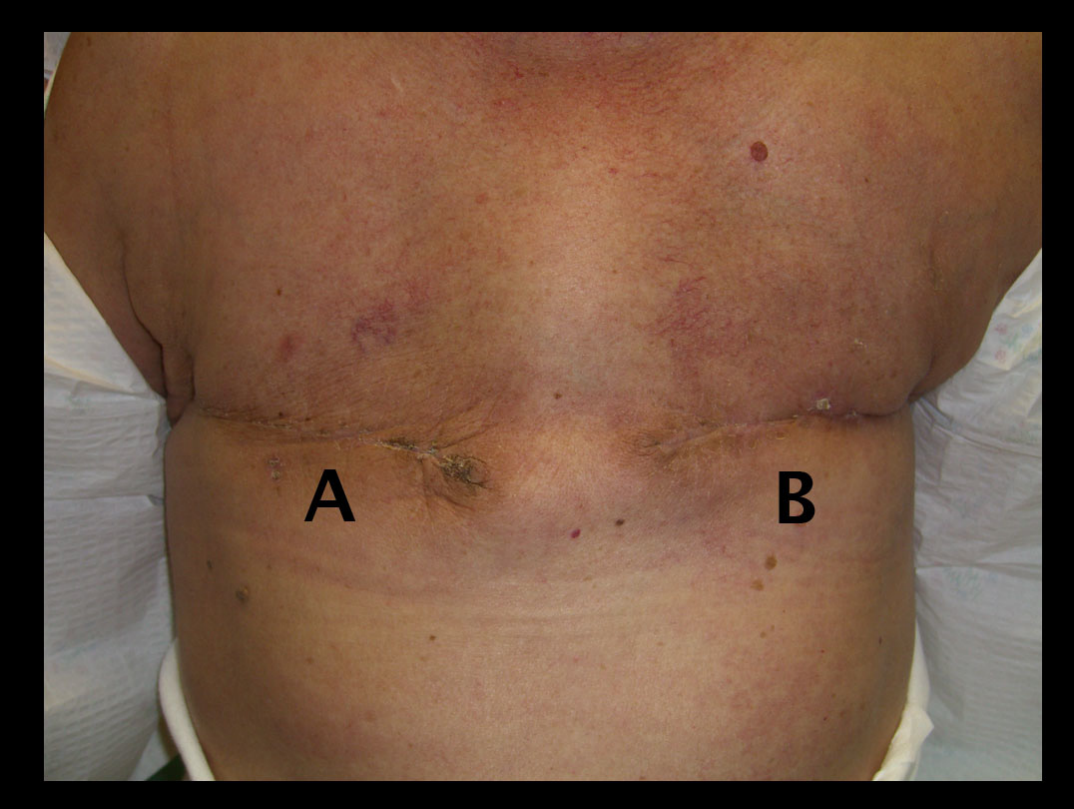
**Healing progress 10 weeks post-mastectomy showing complete healing of both operative sites**.

Intra-operative and post-surgical pathology indicated that the DCIS was non-invasive, with no evidence of disease in the left breast or the right sentinel lymph nodes. The patient was first followed up six days after surgery, and both operative sites were noted to be healing well, with good contour and skin edges approximating as expected (Figure 1). However, at 16 days following surgery, the patient presented with drainage from her incision and an approximately 9cm^2 ^area of necrosis directly superior to the left mastectomy (SOC) incision line (Figure 2). The surgical site on the right side was noted to be clean, dry, and intact. Although the patient was afebrile, amoxicillin-clavulanate prophylaxis was prescribed, and, upon further examination and debridement, the wound was found to undermine in all directions, extending to the sternum and axilla. Subsequently, complete revision and closure of the incision was performed. Following these interventions, the wound healed progressively over the next seven weeks (Figure 3), although a 2cm^2 ^area reopened before healing completely approximately 10 weeks post-operatively (Figure 4).

## Discussion

While malignant tissue resection remains the primary objective of mastectomy, the secondary goals of uncomplicated healing and optimal cosmesis are of paramount importance to the patient. Traditional electrosurgical instruments are widely used in general surgery, chiefly for their efficient bleeding control and dissection capability. However, this surgical efficiency comes at a price: the creation of a deep zone of thermal damage to adjacent tissues that has been associated with delayed wound healing, increased inflammation, and cosmetically unacceptable skin scarring [11-15]. Although the use of traditional devices is acceptable for the majority of surgical procedures, the inherent thermal damage profile may outweigh any potential benefit in select procedures and patient groups. Specifically, during flap development in skin- and nipple-sparing mastectomy, this collateral tissue damage may inadvertently destroy enough of the superficial vascular supply to impair skin flap viability [1].

On the basis of infrared analysis, it has been demonstrated that the cutting surface of the PlasmaBlade operates at a temperature between 40°C and 100°C, compared to 250°C to 350°C for traditional electrosurgical electrodes [9]. Consistent with this finding, pre-clinical and clinical models of cutaneous wound healing have shown that use of the PlasmaBlade enables the surgeon to incise tissue with the precision of a scalpel and the bleeding control of traditional electrosurgery, but with reduced thermal damage that results in improved wound-healing dynamics [9,10,16,17]. The results of the case reported here, in which the differential healing of mastectomy wounds treated with the PlasmaBlade and traditional electrosurgery was observed, are consistent with these previous findings. We hypothesize that unappreciated thermal damage to the subcutaneous microvasculature of the control treatment side in a patient with a noted history of tobacco use may have contributed to significant skin flap necrosis and delayed healing.

## Conclusion

The use of low thermal injury electrosurgical instruments for subcutaneous dissection may help to reduce the risk of skin flap necrosis following mastectomy in select patient groups. In addition, more durable investigations examining this specific hypothesis should be undertaken.

## Patient's perspective

"I had such a hard time with the left side. The pain was terrible. I was horrified and very depressed. My left side is still sore. My right side is perfect, no pain at all. I had expected and hoped both sides would have been as good as the right side."

## Abbreviations

DCIS: ductal carcinoma *in situ*; SOC: standard of care (scalpel and electrosurgery).

## Consent

Written informed consent was obtained from the patient for publication of this case report and any accompanying images. A copy of the written consent is available for review by the Editor-in-Chief of this journal.

## Competing interests

REF is a consultant to PEAK Surgical, Inc. JGV is the Medical Director of PEAK Surgical, Inc.

## Authors' contributions

REF is the principal investigator for the clinical trial described in this report. He performed the mastectomy procedures, conducted patient follow-up, and was a major contributor to the writing of the manuscript. JGV was a major contributor to the design of the clinical trial and to the writing of the manuscript. Both authors read and approved the final manuscript.
